# Novel Papain-Elastase Induced Murine Model for Infrarenal Abdominal Aortic Aneurysm Rupture

**DOI:** 10.21203/rs.3.rs-7166564/v1

**Published:** 2025-08-13

**Authors:** Mohamed Zayed, Santiago Elizondo-Benedetto, Batool Arif, Mohamed Zaghloul, Ibrahim Kuziez

**Affiliations:** Washington University in St. Louis; Washington University in St. Louis; Washington University in St. Louis; Washington University in St. Louis; Washington University in St. Louis

## Abstract

Abdominal aortic aneurysm (AAA) rupture leads to high morbidity and mortality. Current rodent models struggle to reliably mimic infrarenal AAA rupture. Chemical treatments using porcine pancreatic elastase (PPE), papain (Pap), β-aminopropionitrile (BAPN), and angiotensin II (ANG II) are known to induce AAA in rodents. We hypothesized that combining these agents could establish reliable chronic AAA and acute rupture models, resembling human pathology. Here AAAs were induced in male C57BL/6 mice using peri-adventitial exposure to PPE, Pap, or a combination (PPE + Pap), with or without 0.3% BAPN and ANG II. Two weeks post-induction, Pap and PPE + Pap showed increased aortic diameters, higher inflammation, elastase degradation, and matrix metallopeptidase (MMP) activity. Addition of BAPN resulted in large chronic AAAs (500% growth) and intraluminal thrombus (ILT) formation. ANG II-treated mice exhibited a 93% rupture rate, increased inflammation, MMP activation, and ILT formation. These novel murine models are ideal for investigating AAA pathophysiology and therapeutic discovery.

## Introduction

Abdominal aortic aneurysm (AAA) is a life-threatening vascular condition characterized by the localized dilation of the abdominal aorta, which can progress to rupture leading to fatal events.^1,2^ Despite advances in clinical management, AAA rupture continues to be a leading cause of mortality, resulting in an annual mortality rate of approximately 150,000 worldwide.^3,4^ The pathophysiology of AAA involves intricate biomolecular processes, with inflammation playing a crucial role in its progression.^2,5^ Inflammatory cells, such as macrophages and T-lymphocytes, infiltrate the aortic wall and release proteolytic enzymes, including matrix metalloproteinases (MMPs), which contribute to the degradation of the extracellular matrix (ECM).^6–9^ This inflammatory response, often triggered by risk factors such as hypertension and smoking, disrupts the balance between proteolytic activity and ECM synthesis, leading to progressive dilation of the aorta.^10^ However, the exact mechanisms underlying AAA progression and rupture remain unclear, highlighting the need for reliable pre-clinical models to study disease signaling mechanisms and evaluate potential novel therapies aimed at preventing AAA progression and rupture.

Genetically-modified murine models have become essential for investigating AAA pathophysiology.^11–13^ These models aim to replicate key features of human AAA disease, such as aortic dilation, ECM degradation, intraluminal thrombus (ILT) formation and inflammation. Widely used models include genetic variants like *Apoe*^−/−^ mice and chemically induced models. In these traditional mouse models, chemical induction of AAAs can be achieved by using porcine pancreatic elastase (PPE; a proteolytic enzyme that degrades elastin)^14^, β-aminopropionitrile (BAPN; a non-specific inhibitor of lysyl oxidase that disrupts collagen cross-linking)^15^, and angiotensin II (ANG II; a peptide hormone that induces hypertension).^16,17^ Recently it was observed that papain, a cysteine protease derived from papaya latex that also degrades elastin, can also induce AAA formation in rodents.^18^ Although these models offer valuable insights into the molecular and cellular mechanisms of AAA disease, they often fail to fully recapitulate the complexity of human AAAs. This includes challenges in accurately representing body-adjusted AAA diameter, chronicity, aortic anatomical location, and rupture incidence. For example, on their own PPE and papain only induce localized elastin degradation, and fail to replicate the systemic inflammatory and hemodynamic components that resemble human AAA disease progression^14,18^. In contrast, the Ang II model primarily causes aneurysms in the suprarenal aorta, and fails to replicate the infrarenal AAA disease pathology. Moreover, the reliance on hyperlipidemic mice, like *Apoe*^−/−^ mice, limits the applicability of these models to normolipidemic AAA patients, which is a significant drawback.^11,16^

A major challenge in AAA investigations is the failure to translate preclinical findings into effective clinical therapies. Despite the promising results of numerous pharmacological agents in established mouse AAA models, none have progressed to FDA treatments for AAAs in humans.^19^ These translational gaps may be attributed to the lack of standardized protocols for inducing and evaluating AAAs in mice, and inadequate disease modeling of human pathology, which unfortunately causes variability in experimental outcomes.^20^ Therefore, establishing a standardized animal model that closely resemble human AAA disease pathology is crucial for understanding the impact of possible future therapies and improve translational success in humans. In this study we hypothesized that a unique combination of AAA-inducing chemicals can establish reliable and consistent murine AAA models that resemble human chronic disease and acute rupture.

## Results

### Topical PPE and papain exposure promotes aneurysm growth and significantly impacts histopathology and inflammatory markers

**Figure 1:**
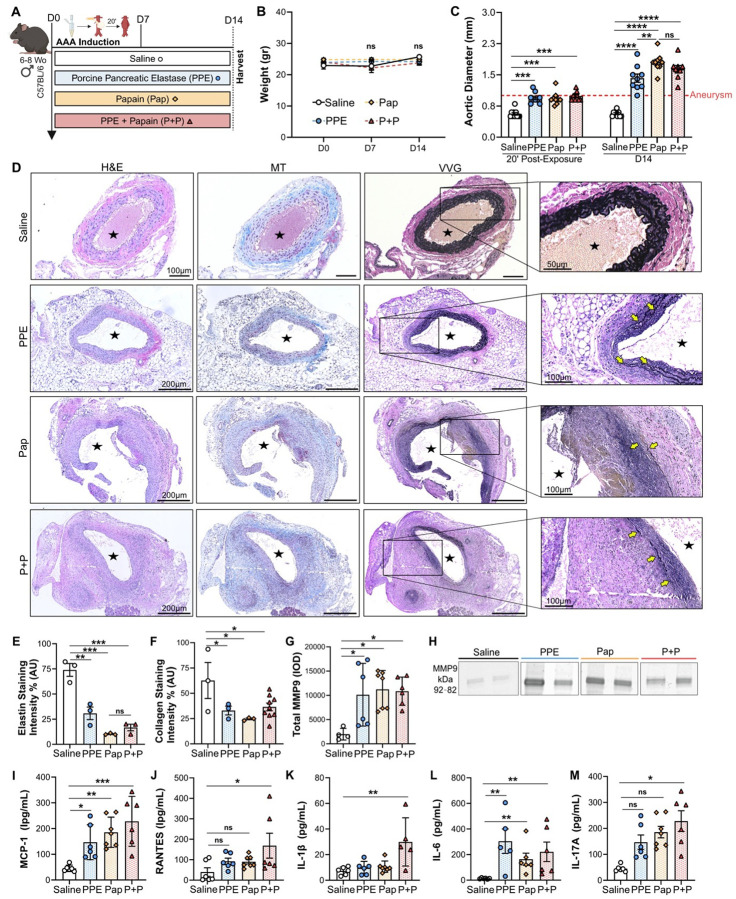
Day 14 AAA formation following peri-adventitial aortic exposure to papain-elastase. (**A**) Mice underwent exposure to either saline (N=10), PPE (N=9), Pap (N=13) or PPE+Pap (N=17) to assess AAA formation. (**B**) Body weight assessment in grams at days 0, 7 and 14 post-AAA development. (**C**) Aortic diameter evaluation in mm, at day 0 (20 minutes post-exposure) and at day 14 (post-AAA development). Aneurysms were defined by a size greater than 1 mm (50% increase from baseline measurements). (**D**) H&E, MT and VVG staining of abdominal aortas (cross-section of tissue slides) with 5x and 10x magnification. (**E**) Quantification of AAA elastin and (**F**) collagen fibers via staining intensity (arbitrary units, AU). (**G**) Quantification of total MMP-9 levels via integrated density (IOD). (H) Zymogram demonstrating total MMP-9 activity band. Chemokines (I) MCP-1 and (J) RANTES and pro-inflammatory markers (K) IL-1β, (L) IL-6 and (M) IL-17A content within the AAA tissue measured by ELISA. Star indicates the aortic lumen. Yellow arrows indicate the elastin fibers. Data are presented as mean ± standard deviation (SD). Ns>0.005, *p<0.05, **p<0.01, ***p<0.001 using either one-way ANOVA, two-way ANOVA with multiple comparison, or student’s t-test when applicable.

AAA development was initially evaluated over a 14-day period (2 weeks) in four different groups of C57BL/6 mice subjected to a 20-minute peri-adventitial (topical) exposure of the infrarenal abdominal aorta to either saline, porcine pancreatic elastase (PPE), papain (Pap), or a combination of PPE and papain (PPE + Pap), as illustrated in Figure 1A and summarized in Table 1. Mice that received topical exposure with each chemical resulted in comparable weight gain (Figure 1B), indicating no significant surgical complications. Compared to the saline group, aortic diameter increased significantly immediately after 20 minutes of chemical exposure to either PPE, Pap or PPE+Pap (0.6±0.1mm vs 0.9±0.1mm, 0.9±0.1mm or 1.0±0.1mm respectively, p<0.001; Figure 1C and **Extended Figure 1**). Furthermore, aortic diameter continued to grow over a 14-day follow-up in the PPE, Pap and PPE+Pap groups (1.4±0.3mm, 1.8±0.2mm and 1.6±0.2mm respectively) compared to saline group (0.6±0.1mm, p<0.0001; Figure 1C and **Extended Figure 1**). Notably, the Pap group exhibited the greatest increase in AAA diameter at week 2, which was significantly higher than in the PPE group (p = 0.002; Figure 1C).

Histopathological characterization was performed using hematoxylin and eosin (H&E), Verhoeff-Van Gieson (VVG), and masson trichrome (MT) stains, as previously described.^21^ All chemically induced groups showed classic AAA dilation with moderate to severe inflammation and elastin degradation, particularly in the Pap and PPE+Pap groups compared to the saline group (Figure 1D and **Extended Figure 2 A-C**). However, vascular smooth muscle cell (VSMC) loss was only mild to moderate in this cohort of mice (**Extended Figure 2D**).

Quantification of VVG and MT staining, conducted as previously described,^22^ demonstrated significant elastin fiber breakdown in the aortic wall of the PPE, Pap and PPE+Pap groups compared to saline group (p<0.001; Figure 1D&E). Additionally, a significant reduction in collagen fibers was observed in the experimental groups compared to the saline group (p<0.01; Figure 1D&F).

Gelatin zymography revealed a significant increase in total MMP9, known to promote AAA formation and rupture,^23^ in the PPE, Pap and PPE+Pap groups compared to the saline group (p=0.03; Figure 1G&H). Moreover, the pro-inflammatory chemokine monocyte-chemoattractant protein 1 (MCP-1), typically linked to AAA inflammation, was significantly elevated in the aortic tissue of the PPE, Pap and PPE+Pap groups compared to the saline group (p = 0.04, 0.003 and 0.0003 respectively; Figure 1I). Interestingly, RANTES levels were significantly increased only in the PPE+Pap group compared to saline group (p = 0.03; Figure 1J). Crucial pro-inflammatory cytokines such as IL-1β, IL-6 and IL-17A were significantly elevated in the aortic tissue of the PPE+Pap group compared to the saline group (p=0.002, 0.002 and 0.0003 respectively; Figure 1K–M). While the PPE and Pap groups also showed significantly increased levels of IL-6 (p= 0.002 and 0.004 respectively; Figure 1L), they did not exhibit significant increases in IL-1β and IL-17A compared to saline controls (Figure 1K and 1M). Additional cytokines, such as IL-10 and TNF-α, showed a slight increase in the PPE+Pap group (p=ns), whereas IFN-y levels were slightly decreased in all three groups compared to the saline group (p=ns; **Extended Figure 2E-G**).

### β-aminopropionitrile enhances impact of papain-elastase in a novel chronic infrarenal AAA model

**Figure 2:**
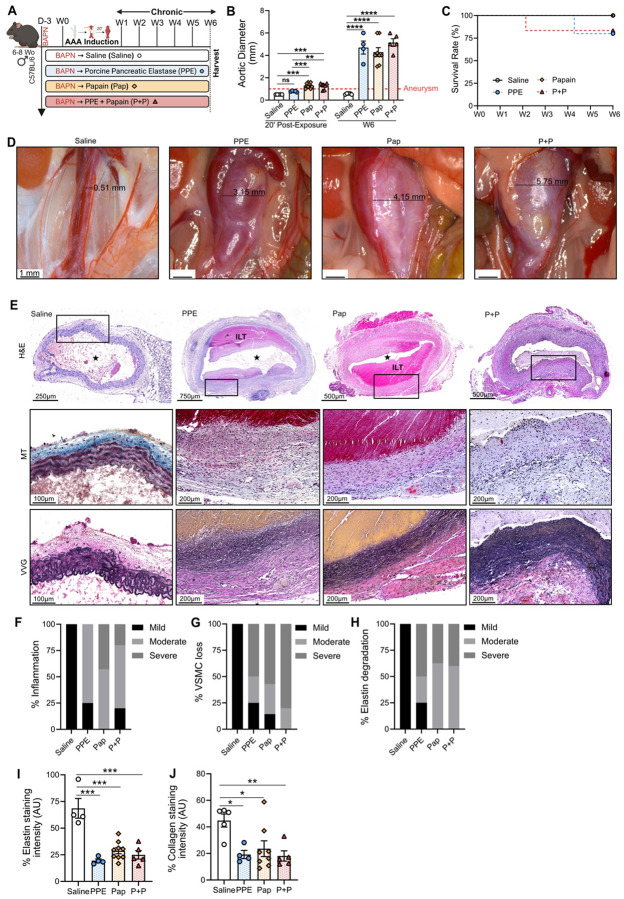
Chronic model of AAA progression using daily BAPN administration over 6 weeks. (**A**) Mice received BAPN through drinking water starting 3 days prior to AAA induction and continuing until week 6. Mice were also exposed to either saline (N=5), PPE (N=5), papain (N=8) or PPE+Pap (N=6) to promote AAA development. (**B**) Aortic diameter evaluation in millimeters at day 0 (20 minutes post-exposure) and at day 42 (post-AAA development). Aneurysms were defined by a size greater than 1 mm (50% increase from baseline measurements). (**C**) Kaplan-Meier curve demonstrating rate of survival following AAA induction. (**D**) Variable impact of chemically induced AAA development on aortic diameter 6 weeks post-AAA development (**E**) H&E, Masson trichrome (MT) and VVG staining of abdominal aortas (cross-section of tissue slides) with 5x and 10x magnification. The star represents the lumen of the artery. ILT = Intraluminal thrombus. IHC categorical analysis (**F**) Degree of inflammation, (**G**) VSMC loss in percent and (**H**) elastin degradation. (**I**) Quantification of AAA elastin and (**J**) collagen fibers via staining intensity (arbitrary units, AU). Data are presented as mean ± standard deviation (SD). Ns>0.005, *p<0.05, **p<0.01, ***p<0.001 using either one-way ANOVA, two-way ANOVA with multiple comparison, or student’s t-test when applicable.

In another murine cohort, aneurysm progression was evaluated over a 42-day period (6 weeks) following the addition of β-aminopropionitrile (BAPN),^15^ a well-established ECM repair disruptor extensively used in murine models of AAA formation. As described above, mice underwent a 20-minute topical exposure to either saline, Pap, PPE or PPE+Pap combination. To further induce AAA progression, 0.3% BAPN was administered daily in drinking water starting 3 days prior to AAA induction (Figure 2A) and in wild type (WT) mice that received only BAPN without operative AAA induction (**Extended Figure 3A**). None of the mouse groups demonstrated differences in weight gain or BAPN consumption throughout the experimental timeline (**Extended Figure 3B&C**).

Compared to the saline group, aortic diameter significantly increased immediately after 20 minutes of chemical incubation with Pap and PPE+Pap (0.5±0.1mm vs 1.1±0.1mm and 1.1±0.1mm respectively, p<0.001) but not with PPE (vs 0.9±0.1mm, p =ns; Figure 2B). Notably, after 6 weeks of BAPN, the aortic diameter showed consistent growth in the PPE, Pap, and PPE+Pap groups, significantly exceeding that of the saline group (0.6±0.02mm vs 4.7 ± 1.2mm; 5 ± 1.4mm and 5.1 ± 0.7mm, respectively; p<0.0001), representing a fivefold increase over the baseline size of the mouse aorta (Figure 2B&D). Mice exposed to PPE exhibited a 20% rupture rate, while the PPE+Pap group demonstrated a 17% rupture rate, and no instances of AAA rupture in the Pap and saline groups (Figure 2C).

Histopathological characterization revealed typical features of aneurysmal degeneration, with increased inflammation and elastin degradation, like the changes observed in the 2-week cohort (Figure 2E). Conversely, VSMC loss was substantially more severe in the chronic cohort, compared to the saline groups (Figure 2E–H). Quantification of VVG-staining demonstrated significant elastin breakdown in the aortic wall of all tested groups compared to the saline group (p<0.001 Figure 2E&I). Additionally, quantification of MT-staining indicated a significant decrease in collagen fibers in the aortic walls of all tested groups, particularly in the PPE+Pap group compared to saline (p = 0.009; Figure 2E&J). Remarkably, almost half of harvested aneurysms displayed ILT formation, closely mirroring human AAA disease pathology (Figure 2E **and Extended Figure 4**). Recent investigations have shown the development of aneurysmal dissection in the ascending and descending aortas using BAPN in younger mice^24^. Therefore, we evaluated both the thoracic and abdominal aortas of WT mice, after 6 weeks of BAPN exposure and performed histological assessment. We confirmed the absence of off-target development of dissections or thoracic aneurysm in this model (**Extended Figure 3D**).

### A novel combination of papain-elastase, BAPN and ANG II provides a reproducible model of acute infrarenal AAA rupture

**Figure 3:**
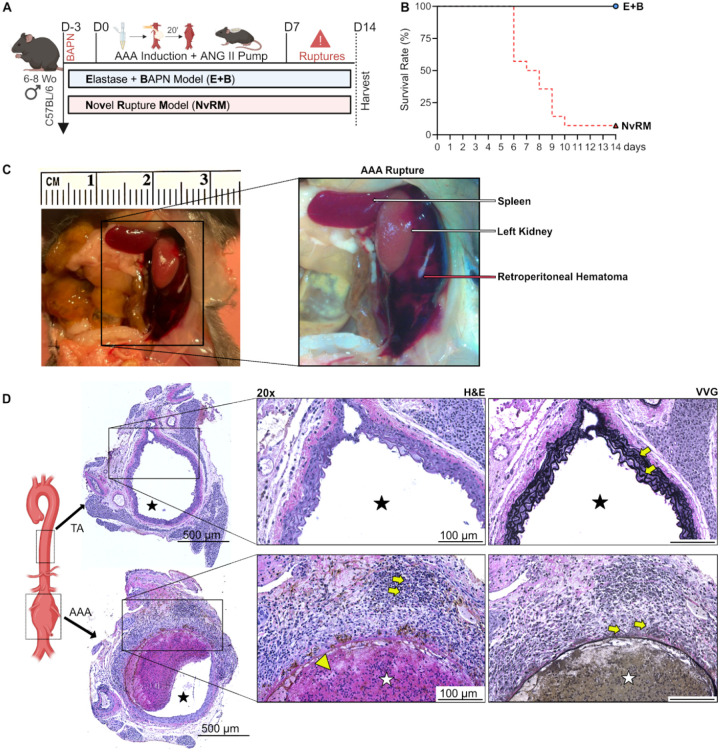
Novel Rupture Model (NvRM) in C57BL Mice. (**A**) Mice underwent exposure to a new combination of papain-elastase (PPE+Pap), BAPN and ANG II subcutaneous pump to assess AAA development and rupture. (**B**) Kaplan-Meier curve demonstrating rate of survival following AAA induction. 93% (13/14) of the NvRM group of mice developed AAA rupture. (**C**) Representative AAA rupture into the left retroperitoneum, associated organs and retroperitoneal hematoma identified with the arrows. (**D**) H&E and VVG staining of the thoracic and abdominal aortas (cross-section of tissue slides) with 5x and 20x magnification. Star = lumen, yellow arrows = cell infiltration and elastin fibers, Big yellow arrow = intraluminal thrombus formation.

AAA was induced using PPE+Pap and BAPN administration, along with ANG II subcutaneous pump to induce hypertension-induced AAA expansion and acute rupture (Figure 3A and Table 1).^17,25^ Mice subjected to this novel model (NvRM) demonstrated a remarkable acute rupture rate of 93% (13 out of 14 mice), with an average rupture time of 7.4±1.5 days post-AAA induction (Figure 3B). Compared to a previously published model of elastase + BAPN AAA rupture model, the NvRM group exhibited a significantly higher rupture rate (Figure 3B).

Consistent with previous murine models of AAA rupture, post-mortem evaluation of the NvRM group consistently revealed retroperitoneal hematomas, a pathognomonic finding of aneurysm rupture events (Figure 3C). Furthermore, an analysis of the single mouse that survived the NvRM combination up to day 14 showed marked increases cellular infiltration and elastin breakdown in the abdominal aorta compared to the thoracic aorta from the same mouse (Figure 3D). The presence of an ILT further closely mirrored human AAA disease pathology (Figure 3D).

### Novel rupture model reveals increased inflammatory signaling and AAA histopathological features prior to rupture

**Figure 4:**
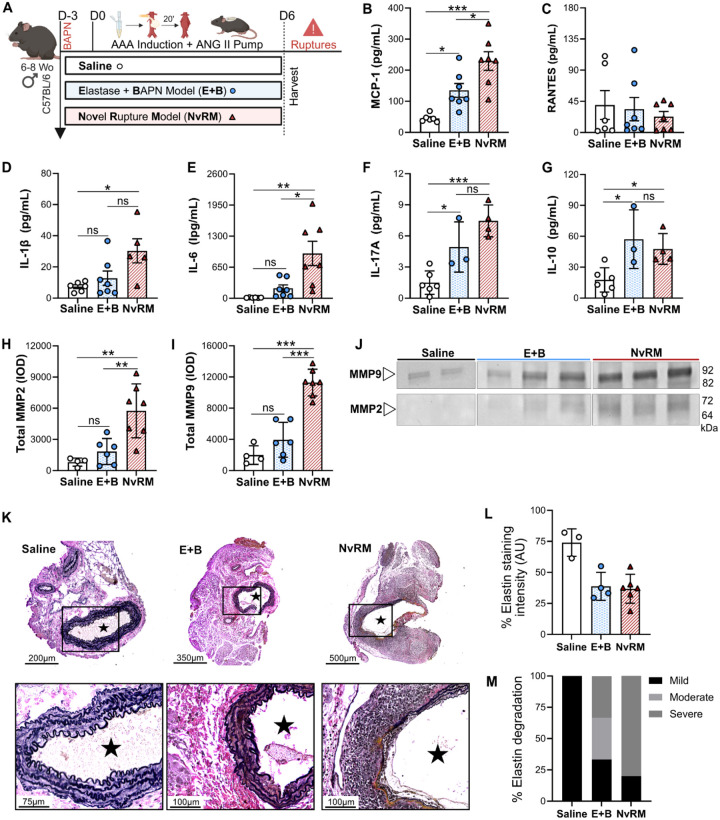
Impact of inflammation and matrix metalloproteinases at day 6 in NvRM. (**A**) Mice underwent exposure to either saline, elastase and BAPN (E+B) or papain-elastase (PPE+Pap), BAPN and ANG II subcutaneous pump (NvRM), to assess AAA inflammation and matrix metalloproteinases at day 6, prior to AAA rupture events. Chemokines (**B**) MCP-1 and (**C**) RANTES and pro-inflammatory markers (**D**) IL-1β, (**E**) IL-6, (**F**) IL-17A and (**G**) IL-10 content within the AAA tissue measured by ELISA. (**H**) Quantification of total MMP-2 and (**I**) total MMP-9 levels via integrated density (IOD). (**J**) Zymogram demonstrating total MMP-9 and MMP-2 activity bands. (**K**) VVG staining, (**L**) quantification and (**M**) qualitative analysis of the abdominal aortas (cross-section of tissue slides) with 5x and 20x magnification. Star = lumen. Data are presented as mean ± standard deviation (SD). Ns>0.005, *p<0.05, **p<0.01, ***p<0.001 using either one-way ANOVA or student’s t-test when applicable.

To investigate the potential pathophysiological factors associated with the high acute rupture rates observed in the NvRM, aortic tissue was harvested on day 6 prior to acute AAA rupture events. Comparisons were made relative to elastase + BAPN (E+B) mice as a positive control and saline treatment as a negative control (Figure 4A). Consistently, MCP-1 chemokine levels were markedly increased in the AAA tissue of the NvRM, compared to E+B and saline controls (p = 0.03 and p = 0.0002 respectively; Figure 4B). The E+B group also demonstrated a significant increase in MCP-1 compared to saline controls (p = 0.04; Figure 4B). RANTES, however, was found to be unchanged among the studied groups (Figure 4C). Pro-inflammatory cytokines IL-1β, IL-6, IL-17A and IL-10 were significantly elevated in the aortic tissue of the NvRM group compared to saline controls (p=0.02, p=0.002, p=0.0004 and p=0.04 respectively; Figure 4D–G). Interestingly IL-6 was also significantly increased compared to the E+B group (p=0.01; Figure 4E). Other cytokines, such as TNF-α, showed a slight increase in both the NvRM and E+B groups (p=ns), while IFN-y was slightly decreased in both groups compared to saline controls (p=ns; **Extended Figure 5A&B**).

Moreover, gelatin zymography demonstrated that total MMP2 and MMP9 activity, which are highly associated with AAA formation and rupture, were significantly increased in the NvRM group compared to both E+B and saline control groups (p<0.01 and p<0.001, respectively; Figure 4H–J) while E+B demonstrated a slight but not significant increase in both MMP-2 and MMP-9 activity compared to controls. Histopathological analysis showed aortic dilation and elevated inflammation at day 6. Importantly, elastin degradation was significantly decreased in both E+B and NvRM groups when compared to controls, but qualitative analysis demonstrated a more severe degradation in the NvRM group (**Figure K-M**).

## Discussion

**Figure 5:**
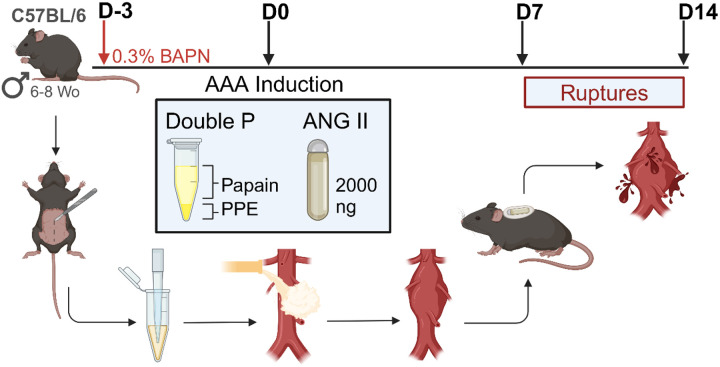
Novel Rupture Model (NvRM) of AAA development and rupture in mice. Synergistic combination of porcine pancreatic elastase (PPE), papain (Pap) β-aminopropionitrile (BAPN) and angiotensin II (ANG II) in C57BL/6 mice. Figure was made using BioRender.com.

In summary, our study demonstrates that the combination of specific factors known to induce aneurysm growth exhibits a synergistic effect on AAA development by enhancing inflammation, MMP activation, elastin fiber degradation, and ILT formation – closely mirroring human disease characteristics.^26,27^ First, we demonstrated that peri-adventitial exposure to a combination of papain and PPE, significantly influences AAA expansion, histopathology, and inflammation over a 14-day period. Secondly, when further enhanced by 0.3% BAPN administration over 42 days, this exposure led to the formation of a chronic AAA, underscoring the model’s utility for long-term studies and distinct inflammatory signals. Lastly, the novel rupture model (NvRM), which combines papain-elastase, BAPN, and ANG II (Fig. 5), achieved a remarkable 93% rupture rate. Moreover, this model exhibited significant elevations in pro-inflammatory cytokines (e.g., MCP-1, IL-1β, IL-6, IL-17A) and MMP activity (MMP2 and MMP9), validating its relevance for investigating AAA progression and potential therapeutic interventions.

In humans, AAAs are most frequently fusiform and develop more often in the infrarenal abdominal aorta, with prior *in vivo* analysis demonstrating less organized collagen fibrils in human aortic tissue in this region.^26–29^ As such, models recapitulating aneurysm formation in this anatomic region are crucial for understanding disease progression and modification. Our model demonstrated a 100% rate of aneurysm development and growth localized to the infrarenal abdominal aorta (Fig. 2D, **Supplementary Fig. 1**), with a predominately fusiform morphology. Additionally, aneurysm ruptures occurred exclusively in the infrarenal abdominal aorta (Fig. 3C). This precise localization is critically important as it mirrors the predominant site of AAA formation and rupture in humans,^28,29^ underscoring the translational relevance and accuracy of our model.

The widely adopted ANG II infusion technique in *Apoe*^−/−^ mice, has been repeatedly demonstrated to lead to aortic ruptures.^16^ While this model replicates hemodynamic and inflammatory changes associated with human AAA and achieves up to a 70% rupture rate when combined with 0.2% BAPN, a limitation of the traditional technique is that aortic ruptures often occur in the thoracic aorta rather than the infrarenal location as seen in the majority of human disease patterns. Also, aneurysms that occur from this model typically result from aortic dissections rather than de novo AAAs.^16,30^ This limits the potential translation to human disease pathology and detailed mechanistic/therapeutic investigations. In contrast, our model not only replicates the widely adopted ANG II infusion and BAPN combination but also achieves a higher rupture rate. Importantly, the aneurysms in our model are uniquely localized to the infrarenal abdominal aorta, with no dissection or tears observed in the thoracic aorta (**Supplementary Fig. 3**). Therefore, the specificity of our model to the infrarenal abdominal aorta provides a more representative and focused approach to studying AAA pathology and interventions.

Moreover, the peri-adventitial elastase model is another widely adopted method that reliably induces infrarenal AAAs and achieves rupture with the addition of 0.2% BAPN.^14,31^ While this model shows consistency and chronicity in AAA development, it falls short in rupture rates, reaching a maximum of 31% in advanced stages.^31^ By replicating this model, we demonstrated a clear superiority of our NvRM (Fig. 3), which demonstrated significantly higher rates of rupture. Additionally, our model showed significantly elevated tissue inflammatory markers, including MCP-1, IL-6, and MMP2 and MMP9 activities – markers extensively linked to AAA disease and particularly prominent in AAA rupture. These markers reached their peak at day 6, just prior to rupture (Fig. 4B, E & H–J). The novel combination of PPE and papain, with a 20-minute abdominal aorta incubation, underscores the efficacy in enhancing AAA progression and rupture pathophysiology.^27^ Our findings highlight the enhanced efficacy of our model in mimicking human AAA rupture pathophysiology, crucial for investigating interventions to mitigate rupture risk.

Lastly, among the spectrum of models designed to induce infrarenal AAA rupture, the use of Transforming Growth Factor-beta (TGF-β) blockade represents another interesting and promising approach.^32^ This model achieves a rupture rate of 40% by inhibiting the TGF-β pathway, which plays a critical role in tissue homeostasis and the inflammatory response. Its potential to reliably mimic AAA in humans is significant. However, adoption by the research community has been limited due to several factors.^32^ Firstly, the high cost associated with *in vivo* TGF-β antibody infusion presents a substantial barrier to widespread use. Secondly, blocking TGF-β – a pathway integral to numerous physiologic cellular processes – raises concerns about off-target effects and its impact on the investigation of therapeutic mechanisms pertaining to AAA rupture. Despite these challenges, the model shows a notable capacity for replicating human AAA conditions, but with complexities that could interfere with comprehensive mechanistic studies. In contrast, our NvRM demonstrates a superior rupture rate of 93%, which is at least 53% higher relative to TGF-β. This significant increase in planned AAA rupture underscores the efficiency and robustness of the model. By avoiding the complexity associated with TGF-β antibody treatment, our approach also allows for a more straightforward interpretation of inflammatory responses and mechanical integrity within the aortic wall.

Another important strength of the NvRM is the presence of ILT formation (**Supplementary Fig. 4**), a distinctive feature of human AAAs that occurs in approximately 70–80% of patients. Recent studies evaluating human AAA biomechanics suggested ILT has a protective role against rupture by reducing strain imposed on the aortic wall.^33,34^ Both our chronic and rupture models exhibited ILT formation (Figs. 2 and 3), facilitating detailed investigations into its role in aneurysm disease progression. ILT is known to contribute significantly to AAA pathology, providing a relevant and translational aspect to our newly characterized murine models.

The models also enabled reliable measurements of various cytokines associated with inflammation in AAA tissue, which are typically linked to disease severity in humans. Inflammatory cytokines, such as MCP-1, IL-6, and IL-1β, play crucial roles in aneurysm tissue and circulation.^35,36^ Recently, our group demonstrated significantly elevated levels of MCP-1, underscoring the importance of the CCR2-MCP1 axis in AAA rupture and highlighting the potential of this pathway for therapeutic investigation.^37^ Additionally, MMP2 and MMP9, were markedly increased in our model, aligning with previously reported data on their role in AAA.^38^ The inflammatory milieu observed in our model closely mirrors that seen in human AAA disease,^35^ reinforcing the relevance of the models.

Several limitations must be acknowledged in our study, which may impact the scope of our findings. Firstly, our study exclusively used male mice. Although AAA prevalence is higher in males, including both sexes in future studies will be essential to assess sex-specific mechanisms and responses in AAA development and rupture. Secondly, the NvRM model exhibited a notably high and rapid rupture rate (93%), which might not fully represent the chronic progression of AAA in humans. While this acute rupture model offers valuable insights into mechanisms leading to rupture, the novel chronic AAA model will provide complementary information related to the slower and more progressive development of AAA. Additionally, our study did not employ *Apoe*^−/−^ mice, typically used to investigate AAAs in the context of atherosclerotic disease. However, applying our models with in *Apoe*^−/−^ mice as well as other specific genetic knockins and knockouts may be vital for future AAA mechanistic investigations. In this context longitudinal monitoring of blood pressure and aortic diameter will be important to facilitate a detailed understanding of the hemodynamic and morphological changes associated with AAA progression. Despite this, the progression of AAA growth was consistent, and the impact of the ANG II infusion on blood pressure has been previously well reported.^16^

## Conclusions

Here we present a novel model that offers a reliable framework for studying AAA rupture in mice and a robust platform for chronic assessment of AAA growth. This model replicates key human AAA characteristics, such as intraluminal thrombus formation and elevated inflammatory markers, enabling reliable measurements of AAA expansion with minimal technical and procedural complications. It is a valuable tool for both acute and chronic AAA mechanistic research. The enhanced inflammatory response and matrix metalloproteinase activities observed underscore its efficacy in mimicking rupture pathophysiology. This specificity to the infrarenal abdominal aorta, coupled with significant elevations in cytokines like MCP-1, IL-6, IL-1β, and MMP2 and MMP9, facilitates detailed investigations into AAA progression and potential therapeutic interventions. Additionally, the chronic model we report provides insights into long-term disease mechanisms, revealing differences in inflammatory signals compared to acute rupture models.

## Online Methods

### Animals

Adult 6–8-week-old, male mice on a C57BL/6 background (n = 117) were obtained from The Jackson Laboratory (Bar Harbor, ME). All animals were housed at 21 °C in a 12/12-hour light/dark cycle and had access to food and water ad libitum. Anesthesia was administered with a mixture of ~1.5% isoflurane and oxygen for all procedures (**Extended Table 1**). The core body temperature was monitored and maintained with a heating pad (37°C). Use of all animal experiments were performed in accordance with relevant guidelines and regulations and approved by the Institutional Animal Care and Use Committee (IACUC) at Washington University School of Medicine in St. Louis. At the conclusion of studies, live animals were euthanized following IACUC protocols.

### Induction of AAA and saline-control models.

**Figure 6:**
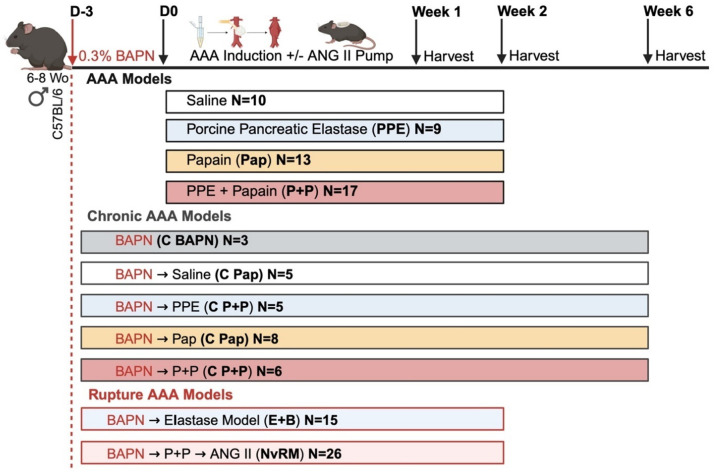
Summary of the methodology to model development to study AAA creation and rupture.

Male mice were induced to develop infrarenal AAAs using either pancreatic elastase^14^(PPE; 10.3mg protein/mL, 5.9 U/mg protein obtained from Sigma Aldrich), papain^18^ (Pap; 1.0 or 20mg/mL) or a novel combination of PPE and Pap (PPE+Pap) while surgical controls were exposed to saline (Figure 1A and 6). Ventral abdominal wall laparotomy was performed, and the infrarenal abdominal aorta exposed, from the left renal vein to the aortic bifurcation. As previously reported, a sterile cotton ball (3.0 × 5.0 mm) was embedded with 50 μL of either PPE, papain or saline using a pipette with a fine tip and placed on top of the aorta to successfully isolate the chemical exposure from the surroundings. After 20 minutes of incubation, the abdominal cavity is washed twice with saline to remove any remnant papain or PPE. The ventral abdomen is then closed in a continuous and interrupted fashion in two layers, with muscle and fascia closed with a 5–0 Vicryl suture, and skin closed with a 5–0 nylon monofilament suture respectively (**Extended Table 1**). Most surgeries were conducted within 30–45 minutes. Using the Leica IVESTA 3 video micrometer, the baseline maximum aortic diameter was measured. After 14 days, all mice aortas were re-exposed *via* ventral abdominal laparotomy, maximal aortic diameters were measured, and aortic tissue was harvested for further analysis. As previously described, aortic aneurysms were defined as >50% increase in the aortic maximum diameter relative to baseline diameter (0.5mm).

### Chronic model of AAA in male C57BL/6 mice

In addition to either Pap or PPE+Pap exposures, starting 3 days before chemical exposure and daily thereafter, two groups of mice also underwent β-aminopropionitrile (BAPN) administration through drinking water (0.3% BAPN in water) to promote AAA rupture^15^. Mice were ≈25 g and drank 2.5 mL water/day, leading to intake of ≈30 mg BAPN/ (kg·day; Figure 2C). After 42 days (6 weeks), all mice aortas were re-exposed *via* ventral abdominal laparotomy, maximal aortic diameters were measured, and aortic tissue was harvested for further analysis (Figure 2A and 6).

### AAA Rupture Induction

In addition to PPE+Pap and BAPN administration, a group of 26 mice underwent a subcutaneous osmotic pump was implanted (Alzet 1004, Durect Corp, Cupertino, CA) to elute angiotensin II (ANG II; Sigma Aldrich Inc, St. Louis, MO) at 2000 ng/kg/min for 14 days as previously described^17,25^, to assess the synergistic effects into a novel rupture model (NvRM) of AAAs (Figure 5 and Table 1). At the 6 or 14-days’ time points, mice were sacrificed, AAA diameters were evaluated, and aortic tissue was harvested for further analysis. Mice that developed ruptured AAAs (RAAA) during the study period promptly underwent necropsy to confirm and analyze the pathology, whereas those animals that did not rupture by day 14 were identified as the non-ruptured AAAs (NRAAA). For merely comparison of the outcomes, a previously described model was replicated using 5 μL PPE topical exposure for 5 minutes. To promote AAA rupture, BAPN administration starting 3 days prior was also assessed in 14 mice (E+B) model as described in Figure 5 and Table 1. Saline-controls mice used in this study were never exposed to BAPN nor ANG II.

### Postoperative Analgesia & Euthanasia

Mice were treated with pre-operative analgesia (Buprenorphine SR) 1 hour prior to the AAA induction procedure. Buprenorphine SR provides 72 hours of pain relief and was dosed at 1 mg/kg and administered subcutaneously. At the time of euthanasia, animals were anesthetized with isoflurane, and the ventral abdominal incision was reopened. The AAA was dissected free from the surrounding tissue, and blood was collected from the inferior vena cava using an insulin syringe (0.33×12.7mm) as summarized in Extended Table 1. The aorta was then excised from the level of the left renal vein to the aortic bifurcation for tissue processing and analysis. The thoracic aorta was also collected and processed accordingly for the studied groups.

### Animal Weight and BAPN consumption

Mice whole body weights were evaluated either at day 0 pre-AAA induction, or from week 1–6 followed AAA induction. Absolute numbers in grams were evaluated and differentiated among studied groups to assess tolerability to the chemical exposure and procedure as well as recovery process. Not significant changes were noted.

### Histology and immunostaining of mice AAA tissue sections.

Aortic tissue was harvested from all animals. AAA tissue was fixed in HistoChoice (VWR), and paraffin embedded. Paraffin blocks were sectioned at 5 μm, and deparaffinized. Processing for antigen retrieval was performed with Sodium Citrate solution, pH 6.0, for 10 min. Tissue sections were blocked with 10% serum, and sections were incubated with primary antibody anti-CD68, 1:100 [Bio-Rad, MCA341GA] and CCR2, 1:200 [Novus-Bio, NBP1–48338]. For Immunofluorescence, sections were incubated with donkey anti mouse (Alexa Fluor 647), and donkey anti rabbit (Cy3) [Jackson ImmunoResearch Laboratories]. A Leica THUNDER Imager 3D was used to acquire the images, which were quantified using Image J Software (NIH) and shown as mean intensity. To evaluate AAA tissue morphology and pathology, tissue sections were evaluated using Hematoxylin and Eosin (H&E), Verhoeff-Van Gieson (VVG), and Mason Trichrome (MT) staining using NanoZoomer (Portsmouth, NH) and analyzed blindly in a semi quantitative manner by a clinical pathologist to assess elastin degradation and VSMC loss within the aneurysm wall.

### ELISA and cytokine array assessments

Proteins were extracted from AAA tissue using RIPA buffer supplemented with a protease inhibitor (Sigma #MCL1). Protein concentrations were determined via the Bradford assay. For each AAA tissue sample, 25 μg of protein was analyzed. Specific assays conducted included a cytokine multiplex assay (Millipore, RECYTMAG-65K), all according to the manufacturers’ instructions.

### MMP2 and MMP9 zymography

For each AAA tissue sample, 25 μg of protein was loaded onto the wells of 10% Gelatin Zymogram electrophoresis gels. The gels were incubated in Zymogram renaturing buffer for 30 minutes, followed by 36 hours in Zymogram developing buffer at 37°C. Subsequently, the gels were stained with Coomassie Brilliant Blue R-25 solution (BioRad) for 30 minutes and then de-stained using a buffer containing 20% methanol, 20% acetic acid, and 60% deionized water until MMP bands became visible. The gels were scanned using a BioRad ChemiDoc system and analyzed with ImageJ software.

### Statistical Analysis

All data are presented as the mean ± SD. Most group comparisons were performed using unpaired t test. For comparisons that included one endpoint in more than one animal/chemically exposed groups, an ordinary one-way ANOVA with multiple comparison was performed. For comparisons that included more than one endpoint in more than one animal/chemically exposed group, a two-way ANOVA with multiple comparison was performed. Data was considered statistically significant with p ≤ 0.05. Kaplan–Meier curve was generated to assess the survival of BAPN-exposed animals. GraphPad Prism 9 (La Jolla, CA) was used for all statistical analyses and graphical data representations. MT and VVG cross section staining’s were analyzed using ImageJ, as previously described.^22^

## Supplementary Material

Supplementary Files

This is a list of supplementary files associated with this preprint. Click to download.

- AAAOperativeVideo7192025.mov
- ExtendedData.docx

## Figures and Tables

**Table 1.** Group models of AAA Development and Rupture

Model	Group	Topical Exposure (μL)	Additional Exposure	Timeline (Days)	Aortic Diameter (mm)*	Rupture Rate
AAA	Saline	Saline (50)	(-)	14	0.6 ± 0.06	0%
	PPE	Elastase (50)	(-)	14	1.4 ± 0.4	0%
	Pap	Papain (50)	(-)	14	1.9 ± 0.2	0%
	PPE+Pap	Papain (50) Elastase (50)	(-)	14	1.6 ± 0.3	0%
Chronic AAA	Sham	No exposure	BAPN	42	0.53 ± 0.06	0%
	Saline	Saline (50)	BAPN	42	0.6 ± 0.02	0%
	PPE	Elastase (50)	BAPN	42	4.7 ± 1.2	20%
	Pap	Papain (50)	BAPN	42	5 ± 1.4	0%
	PPE+Pap	Papain (50) Elastase (50)	BAPN	42	5.1 ± 0.7	17%
Rupture AAA	BAPN+E	Elastase (5)	BAPN	14	1.4 ± 0.4	0%
	NvRM	Papain (50) Elastase (50)	BAPN ANG II	14	N/A**	93%

## Data Availability

The datasets generated and/or analyzed during the current study are available from the corresponding author on reasonable request.
